# Major Differences between Tumor and Normal Human Cell Fates after Exposure to Chemotherapeutic Monofunctional Alkylator

**DOI:** 10.1371/journal.pone.0074071

**Published:** 2013-09-03

**Authors:** Maithili Gupte, Andrew N. Tuck, Vishal P. Sharma, Kandace J. Williams

**Affiliations:** 1 Department of Biochemistry and Cancer Biology, University of Toledo College of Medicine, Toledo, Ohio, United States of America; 2 Amgen, Seattle, Washington, United States of America; 3 Molecular, Cellular and Developmental Biology, University of Michigan, Ann Arbor, Michigan, United States of America; 4 Department of Biological Sciences, Bowling Green State University, Bowling Green, Ohio, United States of America; University of Chicago, United States of America

## Abstract

The major dilemma of cancer chemotherapy has always been a double-edged sword, producing resistance in tumor cells and life-threatening destruction of nontumorigenic tissue. Glioblastoma is the most common form of primary brain tumor, with median survival at 14 months after surgery, radiation and temozolomide (monofunctional alkylator) therapy. Treatment failure is most often due to temozolomide-resistant tumor growth. The underlying basis for development of tumor cell resistance to temozolomide instead of death is not understood. Our current results demonstrate that both cervical carcinoma (HeLa MR) and glioblastoma (U251) tumor cells exposed to an equivalent chemotherapeutic concentration of a monofunctional alkylator undergo multiple cell cycles, maintenance of metabolic activity, and a prolonged time to death that involves accumulation of Apoptosis Inducing Factor (AIF) within the nucleus. A minority of the tumor cell population undergoes senescence, with minimal caspase cleavage. Surviving tumor cells are comprised of a very small subpopulation of individual cells that eventually resume proliferation, out of which resistant cells emerge. In contrast, normal human cells (MCF12A) exposed to a monofunctional alkylator undergo an immediate decrease in metabolic activity and subsequent senescence. A minority of the normal cell population undergoes cell death by the caspase cleavage pathway. All cytotoxic events occur within the first cell cycle in nontumorigenic cells. In summation, we have demonstrated that two different highly malignant tumor cell lines slowly undergo *very* altered cellular and temporal responses to chemotherapeutic monofunctional alkylation, as compared to rapid responses of normal cells. In the clinic, this produces resistance and growth of tumor cells, cytotoxicity of normal cells, and death of the patient.

## Introduction

Standard therapy for glioblastoma is surgery, radiotherapy and temozolomide (TMZ). Clinical trials involving adjuvant therapy to increase patient longevity beyond a median of 14 months have thus far been unsuccessful [1,2]. Treatment failure is primarily due to temozolomide-resistant tumor growth. These clinical results reinforce an important part of the tumor cell arsenal during development of malignancy, which is to develop methods to evade cell death after chemotherapeutic treatment.

**Figure 1:**
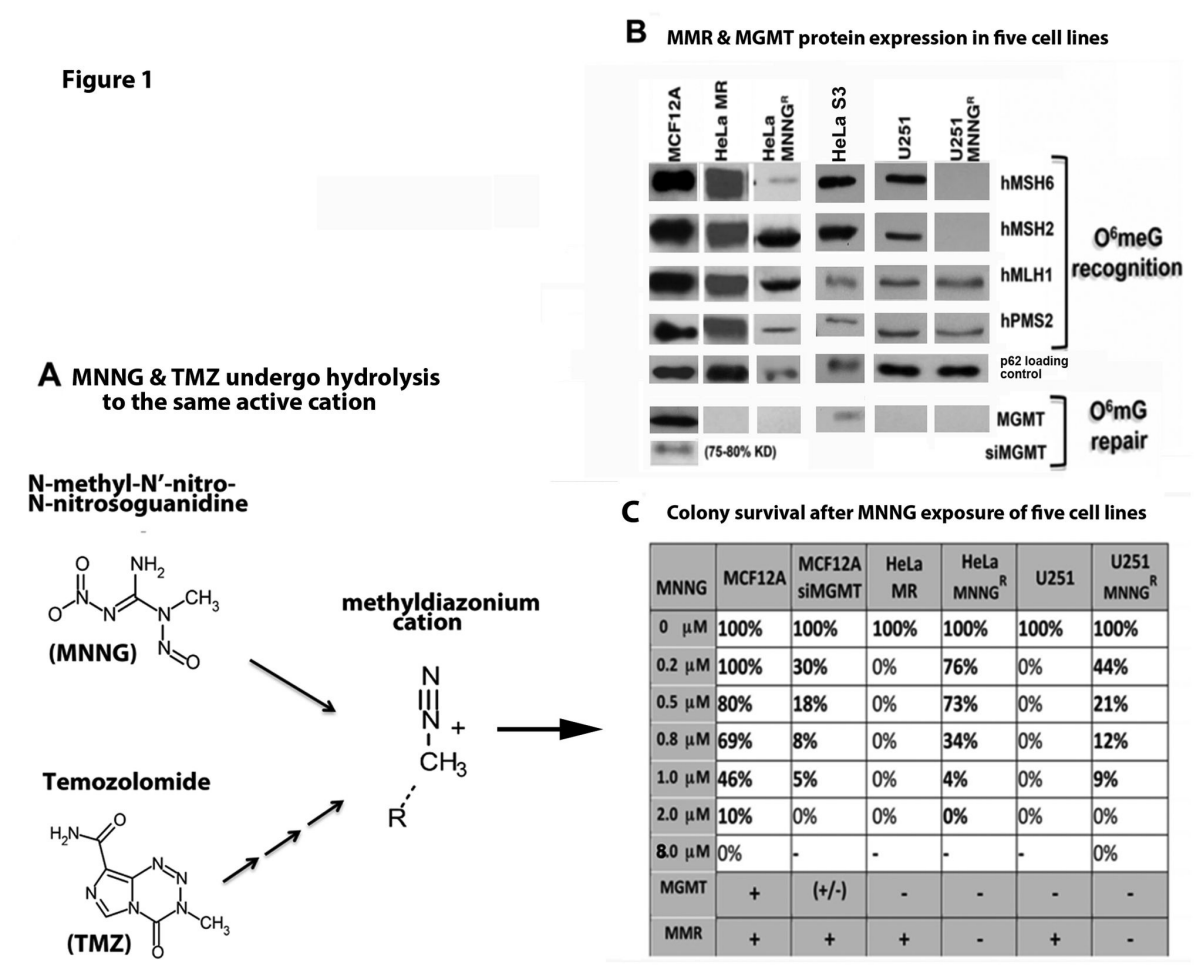
Hydrolysis of MNNG & TMZ to active cation and deficient MMR & MGMT protein expression in cancer cells results in increased colony survival after MNNG exposure. A) *N*-methyl-N’-nitro-*N*-nitrosoguanidine (MNNG) and temozolomide (TMZ) hydrolytic conversion to the same methyldiazonium cation. MNNG requires fewer hydrolytic steps to produce the same highly reactive cation. B) MMR & MGMT protein expression in six cell lines by SDS PAGE and immunoblot and C) colony survival of five cell lines after MNNG treatment. MCF12A (normal mammary epithelium) expressing all four MMR proteins and MGMT are most resistant to MNNG because of MGMT expression. MCF12A +siMGMT are more sensitive because of knocked down MGMT (Figure S1). HeLa MR (cervical cancer) and U251 (glioblastoma) are most sensitive because of lack of MGMT and proficient MMR-induced DDR. HeLa MNNG^R^ and U251 MNNG^R^ are resistant (tolerant) due to lack of both MMR and MGMT. Each colony survival was performed a minimum of four times, with average % survival depicted. SD was less than 5% for each average.

TMZ requires several chemical hydrolysis steps to produce the active methyldiazonium cation. The treatment of cells in culture with *N*-methyl-N’-nitro-*N*-nitrosoguanidine (MNNG) is more reproducible than TMZ, as MNNG requires fewer hydrolytic steps to produce the same highly reactive methyldiazonium cation (Figure 1A). Hydrolysis of both alkylators is independent of enzymatic conversion, and occurs rapidly at physiological pH [3,4]. The methyldiazonium cation forms methyl adducts at several sites on DNA; however the cytotoxicity of TMZ and MNNG is mediated through methylation of the O^6^ position of deoxyguanine (O^6^meG). Base Excision Repair (BER) and Homologous Recombination (HR) efficiently repair all DNA alkylation damage except for O^6^meG, which is directly repaired by methylguanine methyltransferase (MGMT) by covalent attachment of the methyl group from the O^6^meG position to a methyl-acceptor cysteine residue. However MGMT is frequently silenced by promoter hypermethylation in several tissues, such as bone marrow, and up to 75% of high-grade glioblastomas [5–7]. Consequently, cytotoxic activity of monofunctional alkylators is directly attributable to accumulation of O^6^meG, and inversely correlated with expression of MGMT [5,8]. Unrepaired O^6^meG is frequently mispaired with thymine by replicating DNA polymerases that, in turn, activate the noncanonical mismatch repair (MMR)-induced DNA damage response (DDR). This pathway is required for cytotoxic response to the O^6^meG:T mismatched lesion in proliferating cells that lack MGMT. An O^6^meG:T•MutSα•MutLα complex recruits ATR for G_2_/M arrest and subsequent cellular DDR events [9–12]. It is still under debate as to exactly how ATR is activated by O^6^meG:T, but it is it agreed that a proficient MMR system is essential. One model suggests that futile rounds of error-prone mismatch repair opposite the O^6^meG lesion lead to replication fork arrest and DNA breaks, thereby indirectly triggering the ATR DNA damage signaling cascade [13]. A second model provides evidence that binding of O^6^meG:T by mismatch repair proteins directly initiates ATR damage signaling [12,14]. Genetic evidence for this second model has been developed by ‘separation of function’ mutant mice containing mutations in Msh2 or Msh6 ATP processing sequences that are required for canonical MMR but not for MMR-induced DDR. These mice demonstrate that canonical MMR can be destroyed without hindering MMR-induced DDR [15,16]. This second model is emerging as the more important ATR activation pathway. Several investigators have now reported direct interaction between MSH2, ATR and other DDR proteins, but with significant differences from the classical ATR DDR pathway [17–19]. For example, although RPA is required for both canonical MMR and canonical ATR-activated DDR [20,21], RPA is not required for MMR-induced ATR activation [17,19]. Instead, it is believed that MMR proteins act as the scaffold for ATR/ATRIP activation. In agreement with the importance of the MMR pathway to elicit a DDR response to alkylation damage, cells that have proficient MMR and that lack MGMT demonstrate significantly enhanced sensitivity to monofunctional alkylating agents, which correlates directly with initial patient response to TMZ [5,8,22,23]. Conversely, cells that lack both MMR and MGMT are very resistant to cell death, i.e. tolerant, do not undergo cell cycle arrest, and have increased mutation rates. These cells lack the ability to repair O^6^meG lesions due to lack of MGMT expression as the BER pathway does not recognize or repair this lesion. Upon DNA replication, the polymerase frequently misinserts T opposite O^6^mG, creating a mismatched lesion that requires recognition by the MMR pathway for processing [11]. Cells that lack MMR do not recognize this lesion and thus do not give the signal for cell cycle arrest or cell death [24,25]. This response is similar to lack of patient response to further TMZ treatment at the inevitable recurrence of glioblastoma [26–28].

Normal human cells exposed to a sufficient concentration of a DNA damaging agent either undergo stress-induced senescence, or activate the intrinsic caspase cleavage cascade. Stress-induced senescence can be initiated by persistent DNA damage and acts through modulation of the ARF/p53/p21 and/or RB/p16^INK4a^ pathways to arrest mitotically active cells [29–32]. These pathways are frequently lost during carcinogenesis [33–35]. Cells that do not undergo senescence after exposure to DNA damaging agents often undergo programmed cell death (apoptosis). Tumor cells also commonly lack classic apoptotic triggers. The classic caspase cleavage cascade is the most well studied pathway of apoptosis. The two major pathways to caspase activation in human cells are the extrinsic, engaged by death receptors on the cell surface, and the intrinsic, also known as mitochondrial, triggered by DNA damage [36]. Alternatively, Apoptosis Inducing Factor (AIF) is the mediator of a caspase-independent programmed cell death, although little is known in regard to cellular triggers for this noncanonical apoptotic pathway [37]. AIF is cleaved from the inner mitochondrial membrane into the cytoplasm at the onset of mitochondrial membrane permeability (MOMP), after being triggered by cytotoxic events [38,39]. AIF also plays a vital role within the mitochondrial aerobic respiratory chain as an NADH oxidase, which has been functionally separated from AIF death-inducing activity [40]. Activation of nuclear Poly (ADP-ribose) Polymerase-1 (PARP-1) is required for creation of Poly (ADP-ribose) (PAR) polymers to bind and transport the released AIF to the nucleus. Within the nucleus, in collaboration with cyclophilin A and H2AX, AIF binds to DNA to initiate large-scale DNA fragmentation and cell death [37].

Our current studies demonstrate significant differences between normal and cancer cell damage responses to alkylation therapy. These differences include major metabolic, cell cycle, temporal, cytotoxic and death pathways at chemotherapeutic exposure to a monofunctional alkylating agent.

## Materials and Methods

### Cell lines and culture conditions

HeLa MR cervical cancer and U251 glioblastoma cells were grown in DMEM/Ham’s F12 (Invitrogen)/10% fetal bovine serum (Atlanta Biologics, Inc.)/1% Penicillin-Streptomycin (Invitrogen). MCF12A cells (MGMT +) were grown as above, with Mammary Epithelial Growth Supplement (MEGS; Invitrogen) and 50 mg/L Gentamicin at 37°C in a 5% CO_2_ humidified atmosphere. HeLa S3 cells were purchased from ATCC, HeLa MR cells were the kind give of Dr. Sankar Mitra [41]. U251 cells were a kind gift of Dr. William Maltese [42]. MCF12A cells were purchased from ATCC. HeLa MNNG^R^ and U251 MNNG^R^ were developed by plating HeLa MR or U251 cells at 2 x 10^6^ per 150 mm plate, and exposure to a clinical chemotherapeutic equivalent concentration of MNNG (0.2 µM) within 12-16 hr after plating. After one week, this concentration of MNNG yields 0% colony survival for both cell lines, but several individual cells remain alive and attached to the plate [43]. Prolonged incubation (3-4 weeks without additional MNNG) yields several resistant subclones, which are then isolated, grown in complete medium without MNNG, and frozen down. Thawed cells are expanded without exposure to MNNG, then re-exposed to MNNG using the ‘classic’ colony survival protocol (as described below). Reiterative rounds of freeze down, growth and subsequent exposure and subcloning have produced numerous MNNG resistant subclones of HeLa MR and U251 cells. One resistant subclone from each cell line was used for the majority of the experimental studies described in this article.

### Chemicals and reagents

MNNG, thymidine, aphidicolon, staurosporine, HAT media supplement and 6-thioguanine were purchased from Sigma-Aldrich. Propidium iodide was purchased from Invitrogen. Z-Val-Ala-DL-Asp(OMe)-fluoromethylketone (Z-VAD) was purchased from Bachem. 5-bromo-4 chloro-3-indoly A-D-galactopyranoside (X-Gal) was purchased from Sigma and stored at -20°C in the dark as a 40 mg/ml solution in dimethylformamide (DMF). 69 mer oligomers with or without site-specific O^6^meG were purchased from Operon; [α-^32^P]-dATP was purchased from Amersham; Klenow polymerase was purchased from Invitrogen; 4’,6-diamidino-2-phenylindole (DAPI) was purchased from Molecular Probes. Antibodies against MSH2 (NA27), MLH1 (PC56), and PMS2 (NA30) were from Calbiochem; antibodies against MSH6 (610919) and p62 (nucleoporin; N 43620) were from BD Bioscience; antibody against MGMT (NB100-692) was from Novus Biologicals; antibody against AIF (SC-5586) was from Santa Cruz; antibody against GAPDH (MAB374) and nucleophosmin (B23; MAB4500) were from Millipore. Secondary antibodies; Alexa Fluor 488 goat anti-rabbit IgG (green; A21121)) and Alexa Fluor 568 goat anti-mouse IgG (red; A21124)) were purchased from Molecular Probes/Invitrogen. Reagents and protocols for MGMT siRNA knockdown were purchased from Thermo Scientific; Dharmacon RNAi technologies; using ON-TARGET *plus* SMARTpool-Human MGMT (a pool of four proven siRNAs) and DharmaFECT transfection reagent. Mitochondrial metabolic activity was measured as described (XTT Cell Proliferation Assay; ATCC). Apoptosis activity was measured using ApoStat reagents and protocol (R&D Systems). Cellular senescence was determined using the Senescence Detection Kit and protocol from Calbiochem, or by the original assay, as described [44]. Briefly, medium was removed from each 6-well plate and wells were rinsed with PBS, cells were then fixed with 4% buffered formaldehyde at room temperature for 10-15 min. Cells were again rinsed with PBS X^2^ and 1.2 mls fresh staining solution was added to each well (30 mM Citric acid/NaPO_4_ buffer at pH 6.0, 5 mM K_4_Fe(CN)_6_, 5 mM K_3_Fe(CN)_6_, 150 mM NaCl, 2 mM MgCl_2_, and 1 mg/ml X-Gal). Cells were incubated at 37°C overnight in normal atmosphere and examined microscopically the next day for blue-stained cells.

### Protein isolation and immunoblot analysis

Whole cell lysates and nuclear extracts were isolated as described previously [45,46]. After determination of protein concentrations (Bio-Rad), supernatants were stored at -80°C. For immunoblots, equal protein concentrations of whole cell or nuclear extracts were resuspended in SDS sample buffer and separated by denaturing SDS-PAGE. Transfer to polyvinylidene difluoride membrane and immunoblot analyses were performed as previously described [43]. Immunoreactive proteins were visualized by enhanced chemiluminescence following manufacturer’s directions (ECL solution; Amersham Pharmacia Biotech, Inc.) via exposure to X-ray film. Chemiluminescence quantification of each protein band was measured using the Alpha Innotech Fluorochem HD2. Bar graphs and statistics were achieved using Prism GraphPad software. ’***Classic***’ ***colonysurvivalanalysis*** was accomplished by plating 400-600 cells per 60 mm plate and, after cell attachment (12-16 hr), adding the indicated amount of MNNG to each medium. After one week plates were harvested by washing with PBS X^2^, fixing the cells with 100% methanol, and staining with 0.5% crystal violet in 1:1 methanol: ddH_2_O. Colonies containing 50 or more cells were manually counted using a dissecting microscope and the number of surviving colonies on each plate was determined. The average number of colonies from each set of triplicate plates and the percentage survival of each clone were calculated using Microsoft Excel.

### Electrophoretic Mobility Shift Analysis (EMSA)

EMSA was performed as previously described, using equal protein concentrations of nuclear extracts and [^32^P]-end labeled 69mer oligomers and nondenaturing PAGE [45]. ***Hprtmutation* *rates*** for HeLa MR cells and HeLa MNNG^R^ subclones were performed essentially as described [47]. Each HeLa MR population (sensitive and MNNG^R^) was first cleansed of pre-existing *hprt* mutants by growing five successive populations in HAT medium.

### Cell cycle synchronization, DNA damage treatment, and inhibition of apoptosis

Cell cycle synchronization into late G_1_/early S was performed by double thymidine block (DTB) for HeLa MR and U251 cells, as described previously [46]. Immediately after release from DTB and rinse with sterile PBS, 0.2 µM MNNG was added to fresh medium of treated cells. MCF12A cells were synchronized to G_1_/S phase by adding 2 µg/mL of aphidicolon to the medium and incubating for 20 hours. The cells were then rinsed with sterile PBS and released into fresh medium with or without 2 or 8 µM MNNG. To inhibit caspase cleavage-induced apoptosis, Z-VAD (50 nmol/L) was added to U251 cells starting 24 hr after release from DTB and MNNG treatment, and to MCF12A cells 12 hr after release from aphidicolon block and MNNG treatment.

### Cell cycle analyses

Cells subjected to DNA content analysis at specific time points after release from cell cycle block were trypsinized, pelleted by centrifugation (600 x g for 5 min), resuspended in 500 µl PBS, flash frozen in dry ice and stored at -80°C. Cell cycle status at different time points of harvest was determined by measurement of nuclear DNA content by propidium iodide fluorescence using a Beckman/Coulter EPICS Elite flow cytometer, as described previously [43,45]. The resulting data were analyzed by multicycle software (Phoenix Flow Systems)/ modFit LT and reported as the percentage of cells in G_1_, S, or G_2_ phase.

### Indirect immunofluorescence and microscopy

Cells were plated onto glass coverslips at a density of 20,000 cells per coverslip. At the indicated times after cell cycle synchronization and MNNG treatment, cells were fixed with ice-cold methanol for 15 minutes, and then incubated with the indicated primary and secondary antibodies, and DAPI to stain nuclear DNA, as indicated in figure legends, and as described previously [45]. Images were acquired using a Nikon TE2000U fluorescence microscope equipped with a Photometrics Coolsnap EZ Monochrome digital camera system and NIS Elements Basic Research software package.

## Results

### Figure 1. Deficient MMR and MGMT protein expression in resistant cancer cells (HeLa MNNG^R^, U251 MNNG^R^) results in increased colony survival after MNNG exposure.

These studies were initiated to determine if normal human cells and cancer cells have a similar fate, after equitoxic exposure to the monofunctional alkylator MNNG. Both HeLa MR and U251 glioblastoma cells do not express MGMT and therefore exhibit zero colony survival at 0.2 µM MNNG (well within chemotherapeutic range), using the classic colony survival assay (Figure 1B, C). However these colony survival plates have many individual cells still adhered at the time of harvest (1 week). We have previously found that a small subset of the adhered cells develop into resistant colonies after an additional 3-4 weeks of incubation, closely mimicking the inevitable development of TMZ resistance in glioblastoma patients [43]. HeLa S3 and MCF12A cells express MGMT, unlike HeLa MR and U251 parental and subclone cells (Figure 1B). Therefore, we knocked down MGMT in MCF12A cells using siRNA methodology (siMGMT), achieving 75-80% efficiency for up to 96 hr (Figure S1). We then determined equitoxic MNNG concentration for each cell line by assessing colony survival to increasing concentrations of MNNG. Equitoxic concentration for each cell line is defined as the lowest concentration of MNNG at which no colonies survive (0% colony survival). MCF12A cells do not reach 0% colony survival until 8 µM MNNG because these cells express MGMT. Knockdown of MGMT resulted in 0% MCF12A colony survival at 2 µM MNNG. Therefore equitoxic exposure of MNNG to MCF12A cells is at 8 µM and of MCF12a + siMGMT is at 2 µM, as compared to 0.2 µM for both HeLa MR and U251 glioma cells (Figure 1C).

### Figure 2. MMR protein expression and hMutSα activity decrease while hprt mutation rates and MNNG resistance increase in MGMT and MMR negative subclones after repeated exposure to MNNG.

**Figure 2:**
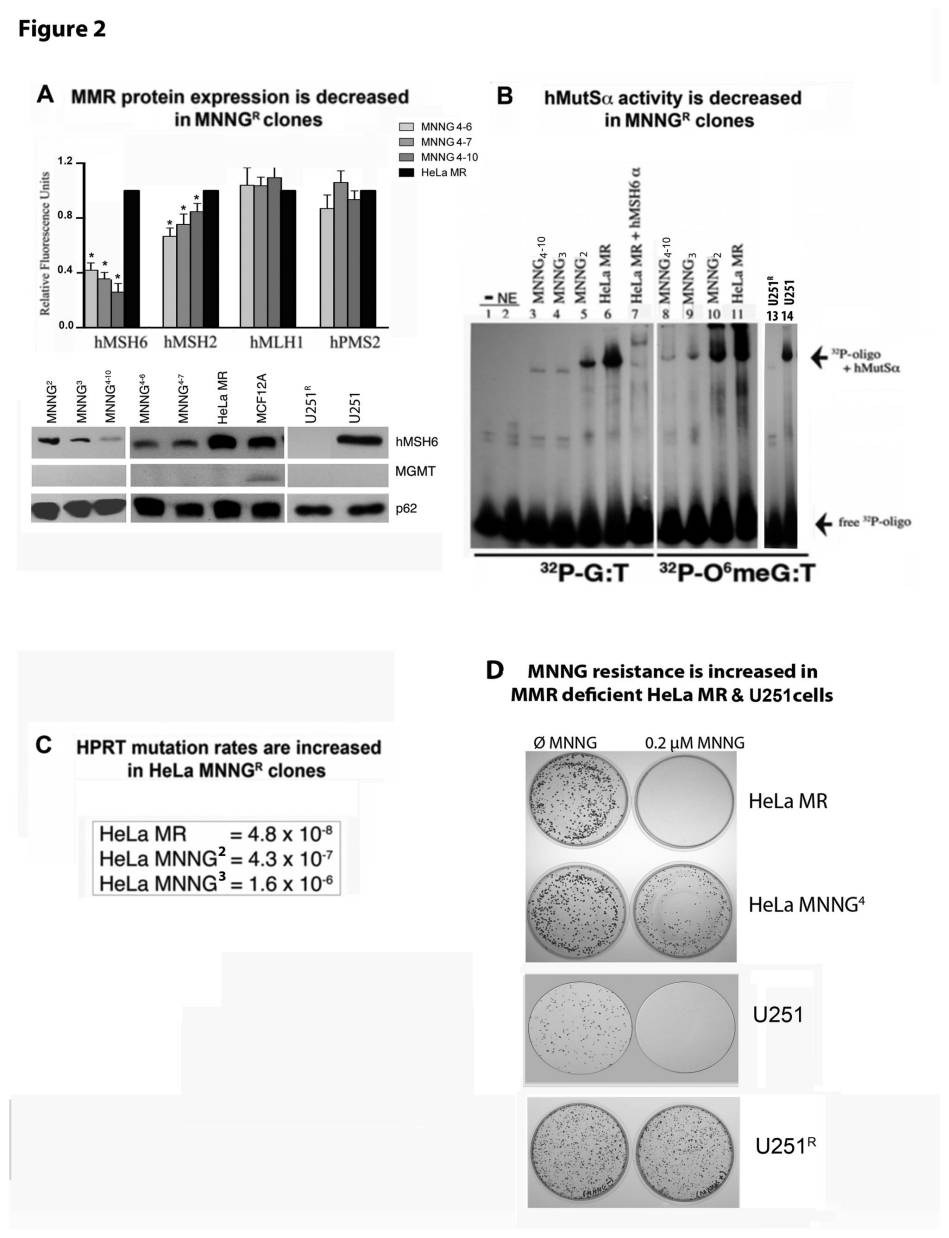
MMR protein expression and hMutSα activity decreases while *hprt* mutation rates and MNNG resistance increases in MGMT and MMR negative subclones after repeated exposure to MNNG. A. MMR protein expression, each fluorescent protein band was measured against a loading control (p62) in the same lane by Alpha lnnotech Fluorochem HD2, histograms produced by Prism GraphPad software, error bars indicate SD. U251 not represented in histogram because hMSH6 and hMSH2 expression is completely absent (Figure 1). Asterisks (*) denote statistically significant differences at P < 0.05 between each subclone protein expression and HeLa MR for the designated MMR protein. Statistical significance determined by student t-test using Prism GraphPad software. Each experiment was performed a minimum of 3 times. B. hMutSα binding activity of equal nuclear protein concentration from nuclear extracts of each cell line by EMSA using [^32^P]-69mer oligomers with either G:T or 0^6^meG:T located in the center. C. *hprt* mutation rates in two sequentially isolated HeLa MNNG^R^ clones as compared to HeLa MR. D. Classic colony survival of HeLa MNNG^4^ and U251^R^ subclones indicates significant resistance to 0.2 µM MNNG as compared to HeLa MR and U251 parental cell lines.

We have thus far developed over 50 subclones of MNNG^R^ HeLa MR and U251 cells, none of which express MGMT (Figure 2 and results not shown). Many of the subclones (but not all) express lower levels of MMR proteins. We selected several HeLa MNNG^R^ subclones and one U251 subclone that exhibited a significant decrease in hMSH2 and/or hMSH6 protein expression (Figure 2A) to determine hMutSα activity by EMSA (Figure 2B). U251^R^ cells exhibited a complete lack of hMutSα, with normal levels of hMutLα (Figures 1B and 2A Western blots, histogram not shown). Each HeLa MR subclone demonstrates decreased binding to both G:T and O^6^ meG:T oligomers. The U251^R^ subclone does not exhibit any mobility shift, as expected. Further, 2^nd^, 3^rd^ and 4^th^ generations of subclones of HeLa MR cells exhibit both decreasing hMutSα activity and increasing mutation rates, as determined by the *hprt* mutation rate assay (Figure 2 B, C) [47]. Finally, colony survival of a 4^th^ generation HeLa MR subclone (HeLa MNNG^4–10^) and the U251^R^ subclone significantly increased after exposure to 0.2 µM MNNG, whereas HeLa MR and U251 parental cell lines exhibited zero colony survival (Figure 2D). The two MNNG^R^ subclones selected for the current studies do not express MGMT, however both subclones have decreased expression of either hMSH6 (HeLa MR MNNG ^R^ {MNNG^4–10^}) or hMutSα (U251 MNNG^R^) (Figures 1B, 2A). In contrast, repeated exposure of MCF12A cells to 8 µM MNNG did not yield altered MMR or MGMT protein levels of the individual surviving cells that were still able to proliferate after several weeks of incubation (Figure S2). We were unable to isolate MNNG^R^ subclones of MCF12A cells using protocol similar to that used for HeLa MR and U251 cells. In summary, repeated exposure of HeLa MR and U251 cells to chemotherapeutic levels of MNNG frequently results in decreased MutSα protein expression and activity, and increased cell survival in cells that do not undergo death. Increased mutation rates observed in the HeLa MR subclones are in agreement with previous literature [25].

### Figure 3. Normal (MCF12A) and cancer (U251 and HeLa MR) cells exhibit very different metabolic (XTT) and cell cycle effects after MNNG exposure.

We have previously noted that the HeLa MR cell cycle is not altered until the 2^nd^ cell cycle after a chemotherapeutic equivalent exposure of 0.2 µM MNNG [43]. Therefore, we decided to investigate if a similar cell cycle response occurred in MCF12A cells at an equitoxic concentration of MNNG (8 µM), as well as in U251 glioma cells (0.2 µM MNNG). We also wanted to determine if cellular metabolic response could be correlated with cell cycle effects.

**Figure 3:**
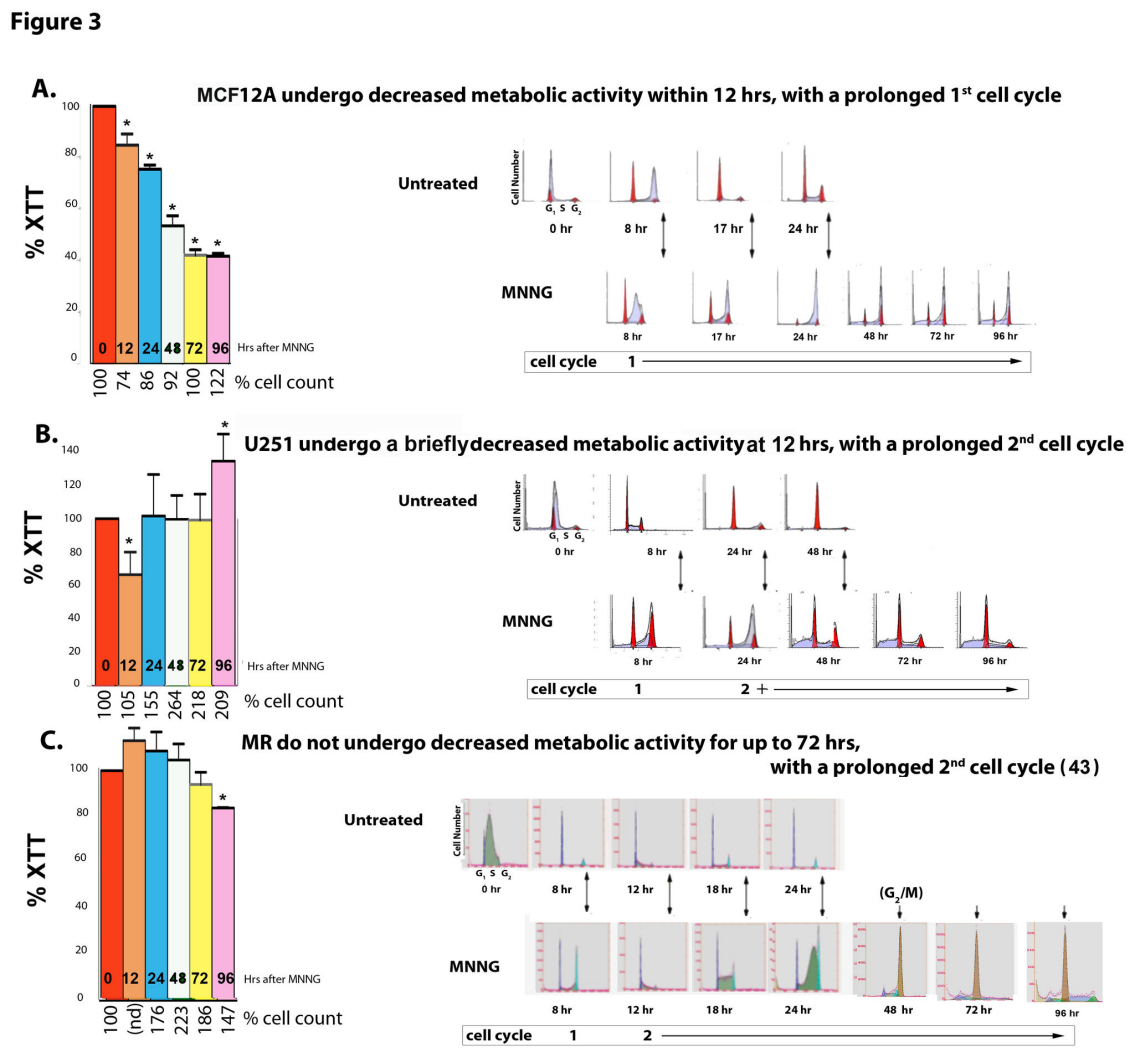
Normal (MCF12A) and cancer (U251 and HeLa MR) cells exhibit very different metabolic (XTT), proliferative, and cell cycle effects after MNNG exposure. All 3 cell lines were subjected to measurements of metabolic activity (XTT histograms produced by Prism GraphPad software, error bars indicate SD), cell counts (% cell count), and cell cycle analysis (flows of DNA content) up to 96 hr post MNNG-treatment (A–C). Each cell line was subjected to an equitoxic concentration of MNNG that resulted in 0% colony survival. Asterisks (*) denote statistically significant differences at P < 0.05 between the metabolic rate measured at that time point and the 0 hr (untreated) metabolic rate of each cell line. Histograms produced by Prism GraphPad software, error bars indicate SD. Statistical significance determined by student t-test using Prism GraphPad software. Each experiment was performed a minimum of 4 times. A. MCF12A normal cells = 8 µM MNNG, B. U251 glioblastoma and C. HeLa MR cell lines = 0.2 µM MNNG. HeLa MR cells cycle events at specific time points after MNNG treatment have been previously published [43].

In contrast to HeLa MR and U251 cells, the majority of the normal MCF12A population does not progress beyond late S phase in the 1^st^ cell cycle after MNNG exposure. In addition, metabolic activity decreases continuously throughout the 96 hr, with the total cell population remaining significantly below the starting population up to 72 hr after treatment (Figure 3A). Metabolic and cell population effects of 2 µM MNNG exposure to MCF12A (10% colony survival) were almost identical to that of 8 µM MNNG, except that metabolism began to recover at 96 hr after treatment (Figure S3). U251 cells pause slightly within the 1^st^ cell cycle, and more significantly within the 2^nd^ cell cycle (Figure 3B). HeLa MRs also pause very slightly in the 1^st^ cell cycle, but go through a very protracted 2^nd^ cell cycle (Figure 3C and previously published in ref [43]). Both cancer cell types continue to proliferate for several days after treatment, and metabolic activities remain at normal levels up to 96 hr post-treatment, except for U251 cells at 12 hr after treatment, unlike MCF12A cells exhibiting a continuously decreasing metabolic rate (Figure 3A, B, C). After 96 hr, both tumor cell populations decline until cell death in all but a very small subset of cells from which eventually grow a much smaller subset of alkylation-resistant colonies [43]. In summary, major differences were measured in cell cycle, cell proliferation and metabolic activity after alkylation exposure to MCF12A, U251 and HeLa MR cells.

### Figure 4. Normal (MCF12A) and cancer (HeLa MR and U251) cells exhibit very different senescent fractions of each population after MNNG exposure.

**Figure 4:**
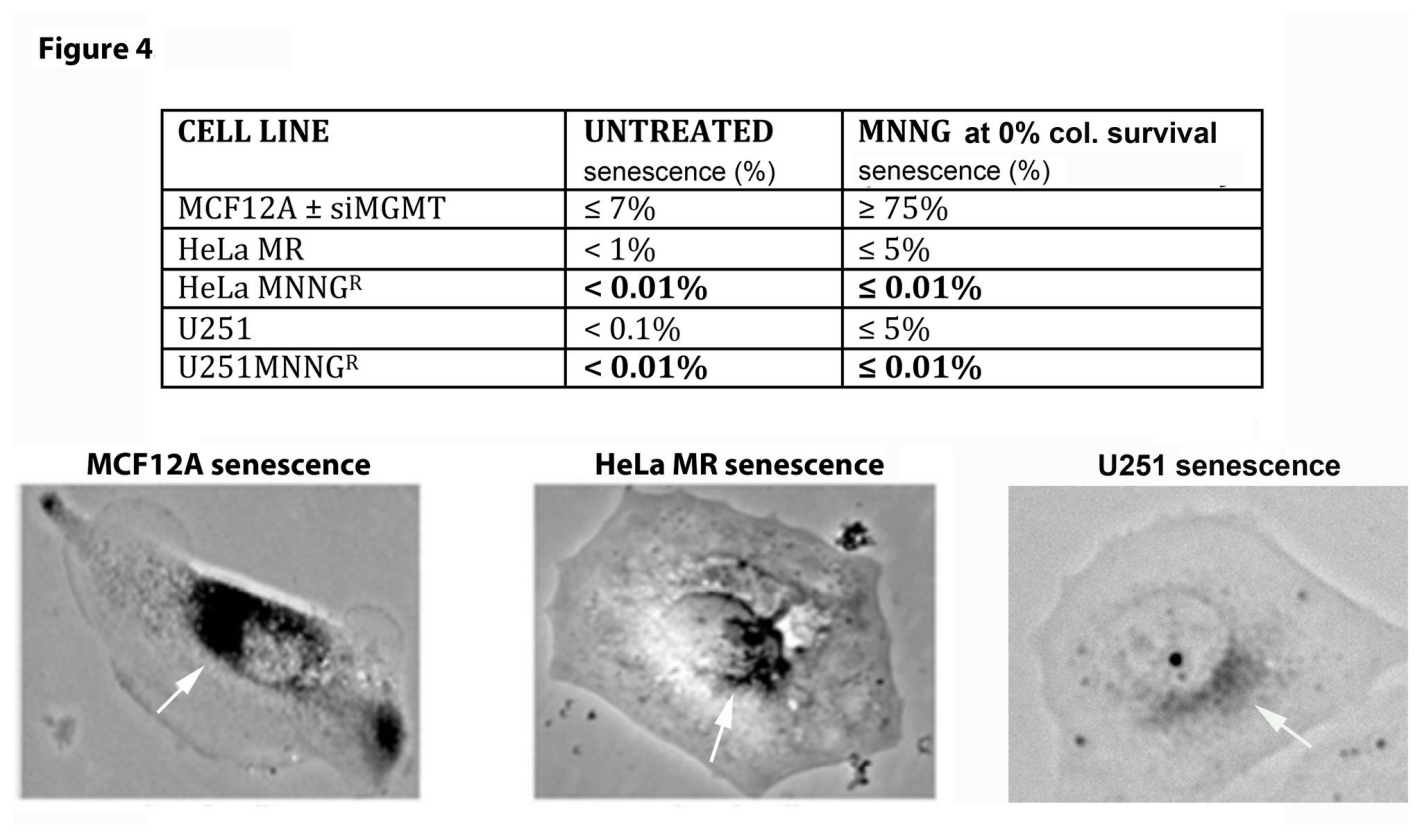
Normal (MCF12A) and cancer (HeLa MR and U251) cell populations exhibit very different senescent fractions after MNNG exposure. Normal (MCF12A) ± siMGMT undergo ≥ 75% senescence at equitoxic concentrations of MNNG (2 µM + siMGMT; 8 µM – siMGMT). Alkylation sensitive tumor cells (HeLa MR & U251) have a low level of ≤ 5% senescence that decreases even further to < 0.01% in alkylation resistant tumor cells (HeLa MR^R^ & U251^R^) at equitoxic concentrations of MNNG (0.2 µM for original cell lines; 2 µM for resistant subclones). Each senescence assay was performed a minimum of 4 times. Light microscopic photomicrograph (150X) of representative senescent cell of each cell line after SA-β galactosidase staining is depicted in lower part of figure, with white arrows pointing to SA-β galactosidase perinuclear stained areas in each cell [44].

Because of the highly altered metabolic activity and cell cycle effects after equitoxic MNNG exposure to MCF12A cells, as compared to the two cancer cell lines, the cell fate of each cell line after MNNG exposure was examined in more detail. Senescence assays were performed of normal and tumor cells exposed to equitoxic concentrations of MNNG. The same protocol as for classic colony survival was used, except plates were initially stained for SA-β galactosidase activity [44] (Figure 4). Although, as expected, no colonies appeared after 1 week, the majority of adherent single MCF12A cells undergo senescence (≥ 75%) after MNNG exposure. HeLa MR and U251 cells exhibit no more than 5% senescence of the individual cells remaining on the plates after MNNG exposure. This decreased senescence of the tumor cells is likely due to the lack of active p53, as p53 binds to HPV 18 E6 expressed in HeLa MR cells [48] and a mutant p53 is expressed in U251 cells [49]. Further, subclones from both cancer cell lines that have developed resistance to MNNG by loss of MMR, undergo even less senescence (< 0.01%) at equitoxic concentrations of MNNG; however, repeated exposure of MCF12A cells to 8 µM MNNG did not alter the rate of ≥ 75% senescence, after four sequential treatments (results not shown). In summary, senescence is the major fate of MCF12A cells, even after repeated exposure to MNNG, however this is not the case with either sensitive or resistant HeLa MR or U251 cancer cells after equitoxic exposure to MNNG.

### Figure 5. Normal (MCF12A) and cancer (HeLa MR and U251) cells exhibit very different caspase cleavage activity after MNNG exposure.

**Figure 5:**
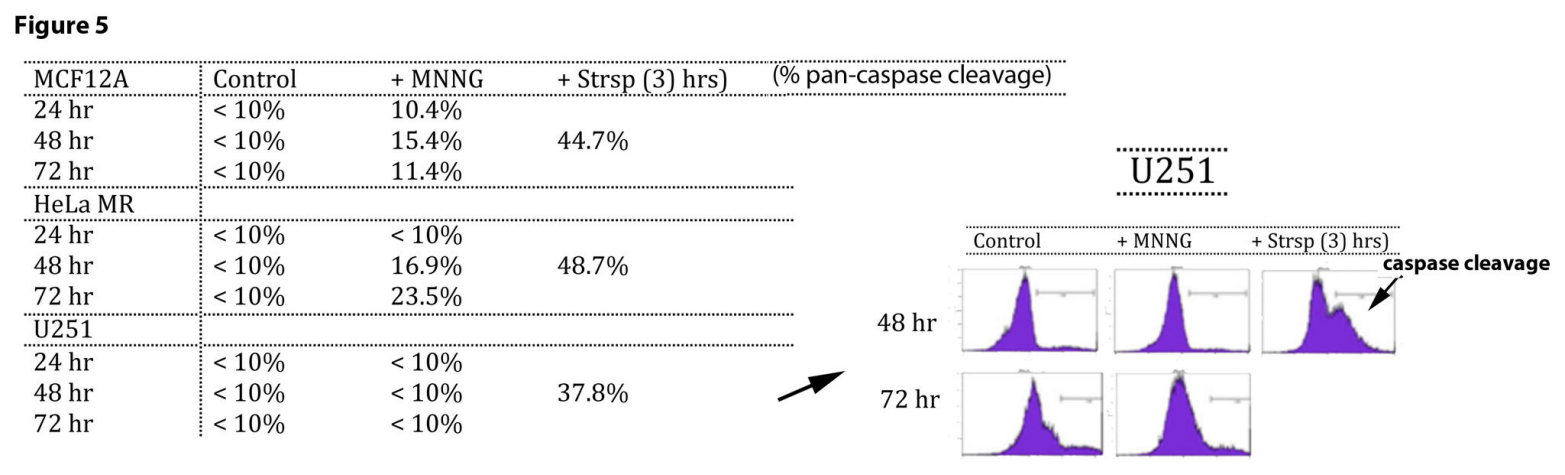
Normal (MCF12A) and cancer (HeLa MR and U251) cells exhibit very different caspase cleavage activity after MNNG exposure. Pan-caspase cleavage detection (ApoStat Apoptosis Detection Kit; R&D Systems) using flow cytometric analysis of a pan-caspase inhibitor can detect total percent of the population containing caspase cleavage products. Both MCF12A and HeLa MR have detectable amounts from 24-72 hr, but not U251 cells after MNNG treatment (<10%) up to 72 hr later. As positive control, caspase cleavage is detected in all cell lines within 3 hr after staurosporine (Strsp) treatment. Each assay was performed a minimum of two times.

In an endeavor to validate the fate of cells not undergoing senescence, a pan-caspase fluorescence detection method was used to measure apoptosis via the classic caspase cleavage cascade (Figure 5). Small fractions of both MCF12A and HeLa MR populations undergo caspase cleavage after MNNG treatment. Caspase cleavage within MCF12A cells is just detectable at 24 hr and peaks at 48 hr at 15.4%, corresponding well with a total senescent population at ≥ 75%. Caspase cleavage in HeLa MR cells is not detectable until 48 hr and is highest at 72 hr at 23.5%. We could not detect caspase cleavage above background in U251 cells for up to 72 hr after MNNG treatment, except in staurosporine-treated populations (positive control for caspase cleavage). Significant caspase cleavage in both tumor cell lines after staurosporine exposure indicates that both cell lines are capable of caspase cleavage events.

Both HeLa MR and U251 cancer cell lines undergo ≤ 5% senescence after MNNG treatment. Therefore, this small amount of senescence combined with the limited extent of caspase cleavage after MNNG treatment does not account for the observed high amount of eventual cell death in these tumor cell populations. In agreement with the above caspase cleavage results, inhibition of caspase cleavage by Z-VAD produces a slight but significant inhibition of cell death in HeLa MR cells at 72 hr after MNNG treatment [43] and also in MCF12A cells at 48 hr, but not in U251 cells up to 96 hr after MNNG treatment (Figure S4). In summary, both senescence and caspase cleavage-induced apoptosis appear to account for the majority of MCF12A cell fate, but neither pathway can account for a significant portion of the cell fate of HeLa MR or U251 cancer cell populations exposed to a chemotherapeutic concentration of MNNG.

### Figure 6. Apoptosis Inducing Factor (AIF) translocates to the nucleus in cancer cells (U251 and HeLa MR) but not in normal cells (MCF12A) after MNNG exposure.

**Figure 6:**
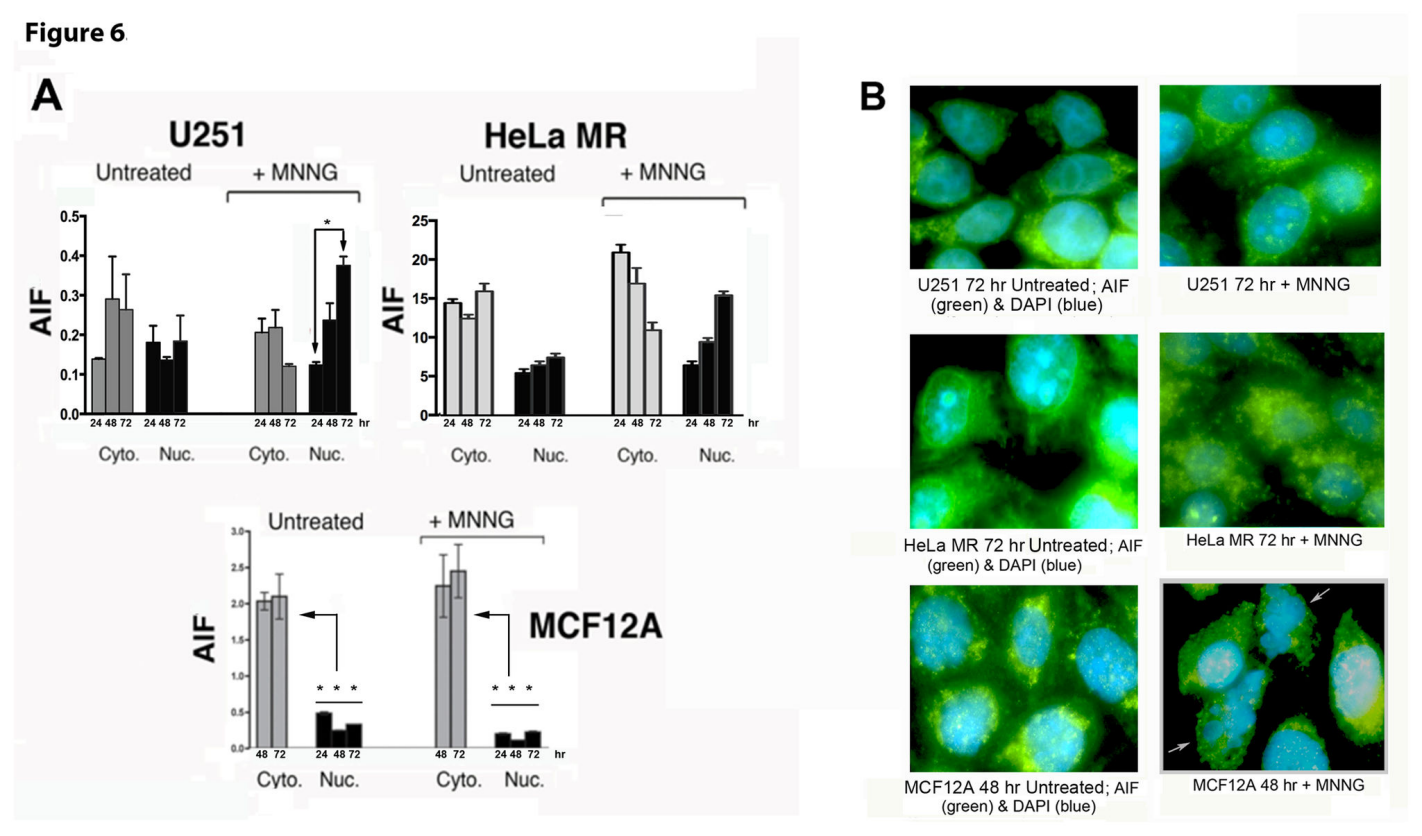
Apoptosis Inducing Factor (AIF) translocates to the nucleus in cancer cells (U251 and HeLa MR) but not in normal cells (MCF12A) after MNNG exposure. U251 and HeLa MR tumor cells exhibit increased AIF traversal into the nucleus 48-72 hr after MNNG (0.2 µM). MCF12A normal human cells do not exhibit increased traversal of AIF into the nucleus up to 96 hr after equitoxic MNNG (8 µM). A. AIF detected by cell fractionation and quantitative immunoblotting. Each fluorescent band was measured against a loading control (cytoplasmic GAPDH or nuclear lamin) in the same lane. Asterisks (*) denote statistically significant differences at P < 0.05 between the amount of AIF measured within nuclear extract at 72 hr and at 24 hr after MNNG treatment (U251 + MNNG), or between each nuclear extract and cytoplasmic extract concentration at 72 hr (MCF12A). Histograms produced by Prism GraphPad software, error bars indicate SD. Statistical significance determined by student t-test using Prism GraphPad software. Each experiment performed a minimum of two times. B. Microscopic immunofluorescence (100X) using AIF antibody (green) and DAPI (blue). HeLa MR and U251 cells exhibit maximum AIF within the nucleus at 72 hr. MCF12A cells do not exhibit AIF traversal to the nucleus, but do exhibit apoptotic nuclei at 48 hr as indicated by the two white arrows in the lower left photomicrograph.

The AIF pathway, a caspase-independent programmed cell death, has previously been implicated in alkylation-induced cancer cell death, therefore we investigated this programmed cell death pathway [40,50]. Indeed, our current studies reveal that activation of the AIF death pathway in HeLa MR and U251 glioblastoma cells occurs with increased AIF translocation to the nucleus, evident by nuclear and cytoplasmic fractionation and quantitative immunoblotting, as well as microscopic immunofluorescence (Figure 6A, B). It is notable that AIF translocation to the nucleus does not become elevated above cytoplasmic concentrations in either cancer cell line until 72 hr after exposure to MNNG. These results are in agreement with peak levels of cytotoxicity previously noted in these cells after MNNG treatment [43]. In contrast, AIF does not translocate to the nucleus in MCF12A cells to any measurable extent up to 72 hr, however apoptotic nuclei are evident at 48 hr after MNNG exposure, correlating with the peak caspase cleavage time point (Figures 5, 6B). Our current results agree with the literature in that caspase-independent cell death often occurs with much slower kinetics and different characteristics than classic caspase cleavage-induced apoptosis [38,51].

In summary, these results demonstrate that chemotherapeutic levels of MNNG-induced AIF cell death in tumor cells is associated with multiple cell cycles, ongoing proliferation, uninterrupted metabolic activity, extremely prolonged time to death, decreased senescence, and eventual growth of resistant subclones that frequently have dysfunctional MMR (Figures 1-6). In contrast, equitoxic concentrations of MNNG cause the majority of normal cells to undergo senescence, and a minority to undergo caspase cleavage-associated apoptosis in the first cell cycle after MNNG treatment, without measurable development of resistant subclones, even after multiple exposures.

## Discussion

Our current studies demonstrate significant differences in chemotherapeutic response between normal human cells and cancer cells. After exposure to a chemotherapeutically equivalent concentration of MNNG, the entire cancer cell population traverses multiple cell cycles and maintains metabolic output for several days. Most of the cells eventually die via the non-canonical Apoptosis Inducing Factor (AIF) pathway, with minimal caspase cleavage or senescence occurring in the treated population. A very small fraction of individual tumor cells survive and approximately 1/50,000 cells eventually resume proliferation [43]. Clones developed from these tumor cells are often permanently resistant to further alkylation exposure. All of the permanently resistant subclones that we have examined thus far do not express MGMT, and in this respect remain unchanged from parental cell lines, both of which do not express MGMT because of promoter hypermethylation. This is not unanticipated: promoter hypermethylation is not likely to be reversed by additional methylation treatment. The overwhelming majority of the resistant subclones have a deficient MMR pathway and therefore lack MMR-induced DDR and cell cycle arrest after re-exposure to MNNG. The few resistant subclones that exhibited normal levels of MMR protein expression (results not shown) may have had point mutations in one or more of the MMR proteins, or may have only been temporarily resistant to MNNG [52]. We are currently investigating these possibilities. Unfortunately, acquired inhibition of the MMR pathway, allowing tolerance to O^6^mG lesions and chemoresistance, has been repeatedly documented in malignant glioblastoma, as well as in alkylation therapy-related leukemias [26–28,53–56]

Conversely, normal human cells arrest within the first cell cycle, even after multiple exposures to equitoxic levels of MNNG. Cellular metabolism plummets within the first 12 hours, likely because the majority of the population undergoes senescence, while a minority undergoes classical apoptosis involving the caspase cascade. Similar results occur at equitoxic MNNG levels in MCF12A cells with knocked down MGMT. Despite repeated attempts, we were unable to isolate MNNG resistant subclones of MCF12A cells. Instead, the few cells that escaped senescence or death were equally sensitive to subsequent MNNG exposures. These results also agree with recent literature in that endothelial cells within the glioblastoma microvasculature primarily undergo senescence after alkylation and irradiation therapy [57]. It is also significant that all of the above cellular effects occurred much more rapidly within normal human cells as compared to cancer cells. Differences in temporal response to alkylation exposure, combined with continued cell cycling and metabolic rate may give tumor cells advantages beyond that of normal cells for development of resistance to chemotherapy. However, the differences in response to MNNG exposure between normal and tumor cells likely indicate several differences in pathways of response that could eventually become therapeutically advantageous.

Overall, steps culminating in the choice between alkylation-induced tumor cell resistance or cell death are not understood. The half-lives of both MNNG and TMZ are very short (≤ 1.5 hr) compared to 4-7 days until cell death [3,4,43]. TMZ and MNNG are not substrates for multidrug transporter reflux at the plasma membrane, nor require metabolic enzymes for hydrolysis to the active moiety [3,58]. At chemotherapeutic levels, alkylation toxicity is targeted to genomic DNA. Maximum tolerated clinical dose of TMZ (2 mM) results in a peak plasma concentration of the methyldiazonium molecule of ≤ 0.5 µM [4,59]. Our results show that HeLa MR and U251 cell lines lacking MGMT exhibit 0% colony survival at 0.2 µM MNNG, well within clinical chemotherapeutic concentration of the active moiety of TMZ. At this concentration, all DNA alkylation damage is repaired efficiently by BER and HR, with the exception of O^6^meG adducts. In proliferating cells that lack MGMT expression, O^6^meG adducts become O^6^meG:T lesions and require MMR-induced DDR to inhibit continued proliferation and mutations at these sites [5,8–11]. Cells also lacking MMR continue to proliferate and suffer increased mutation rates both at lesion-containing sites and throughout the genome because of a lack of MMR proof-reading activity during DNA replication.

BER is virtually never disabled, and therefore has become the target of synthetic lethal anti-cancer therapy, via PARP inhibition, in rare cancers that have a genetic defect in HR (*BRCA 1/2*) [60]. Unfortunately, the majority of these rare HR-deficient tumors develop resistance by acquiring additional mutations that re-establish the HR pathway [61]. The effectiveness of disabling BER by the use of PARP inhibitors for treatment in conjunction with alkylatin therapy for glioblastoma and for other tumors as well is not clear, however several clinical trials are ongoing (http://www.clinicaltrials.gov) [62,63]. Parthanatos, a unique form of PARP-1 mediated cell death, similarly requires PAR transport of AIF to the nucleus after alkylation treatment. During parthanatos, however, AIF release is an early event from an AIF pool on the outer mitochondrial membrane, requiring early and excessive PARP-1 activation and a rapid cell death, likely due to ATP depletion and metabolic starvation. An important point to note is that to achieve parthanatos, 50-500 µM MNNG is required which is 100-1000 times the therapeutic equivalent [50,64,65]. Clearly, our current studies do not elicit a parthanatos reaction in the cancer cells as cellular metabolism is not immediately affected, nor does death occur rapidly.

Our current results, combined with the literature in this field with regard to tumors lacking MGMT expression, support the notion that the majority of tumors lacking MGMT expression also develop MMR pathway deficiency as a primary mechanism of TMZ resistance at chemotherapeutic concentrations, and ultimately causes death of the patient [26–28,53–56]. Notably, we have found major differences between normal and cancer cells in regard to both temporal and cellular pathway response to alkylation chemotherapy, resulting in very different ultimate cell fates. There is still much that we do not understand at the molecular level, as is often the case with clinical chemotherapy. A better understanding of the strategies used by tumor cells to evade cell death, while developing chemotherapeutic resistance, is central to devising more effective therapeutic targets.

## Supporting Information

Figure S1 **MGMT knock down in MCF12A human cells.** MGMT expression was knocked down by 4 different siRNAs against MGMT. Upper figure is immunoblot of MGMT protein expression after loading equal protein concentrations in each lane, up to 96 hr after MNNG exposure with p62 as a loading control. Lower graph is a histogram produced by measurement of each fluorescent MGMT band against the p62 loading control in the same lane by Alpha lnnotech Fluorochem HD2, histograms produced by Prism GraphPad software.
(ZIP)

Figure S2 **Repeated exposure to MNNG does not alter MMR or MGMT expression in surviving MCF12A populations.** MMR protein expression of equal protein concentrations from MCF12A original cell line and from surviving MCF12A cells grown from three sequential MNNG exposures that result in 0% classic colony survival (8 µM).
(ZIP)

Figure S3 **MCF12A cells exposed to 2 µM MNNG regain metabolism by 96 hr after exposure.** MCF12A cells exposed to 2 µM MNNG results in 10% classic colony survival (Figure 1), allowing surviving cells to exhibit increased metabolic activity by 96 hr, although still significantly lower than 0 hr (untreated) control (compare to Figure 3A). Asterisks (*) denote statistically significant differences at P < 0.05 between the metabolic rate measured at that time point and the 0 hr (untreated) metabolic rate of each cell line. Histograms produced by Prism GraphPad software, error bars indicate SD. Statistical significance determined by student t-test using Prism GraphPad software. This experiment was performed two times.
(ZIP)

Figure S4 **Treatment of MCF12A and U251 cells with Z-VAD decreases cell death in MCF12A cells, but not U251 cells.** MCF12A cells exhibit decreased cell death at 48 hr after MNNG treatment (8 µM) by addition of Z-VAD to media. U251 cells do not exhibit decreased cell death at any time point up to 96 hr after MNNG treatment (0.2 µM). Asterisk (*) denotes statistically significant differences at P < 0.05 between the cell count measured at that time point (48 hr) and the 0 hr (untreated) MCF12A cell count. Histograms produced by Prism GraphPad software, error bars indicate SD. Statistical significance determined by student t-test using Prism GraphPad software. These experiments were performed a minimum of three times.
(ZIP)
