# Supplementary figures and images for: Major Differences between Tumor and Normal Human Cell Fates after Exposure to Chemotherapeutic Monofunctional Alkylator
Source: PLoS One. 2013 Sep 3;8(9):e74071. doi: 10.1371/journal.pone.0074071 (PMC3760805; doi:10.1371/journal.pone.0074071)

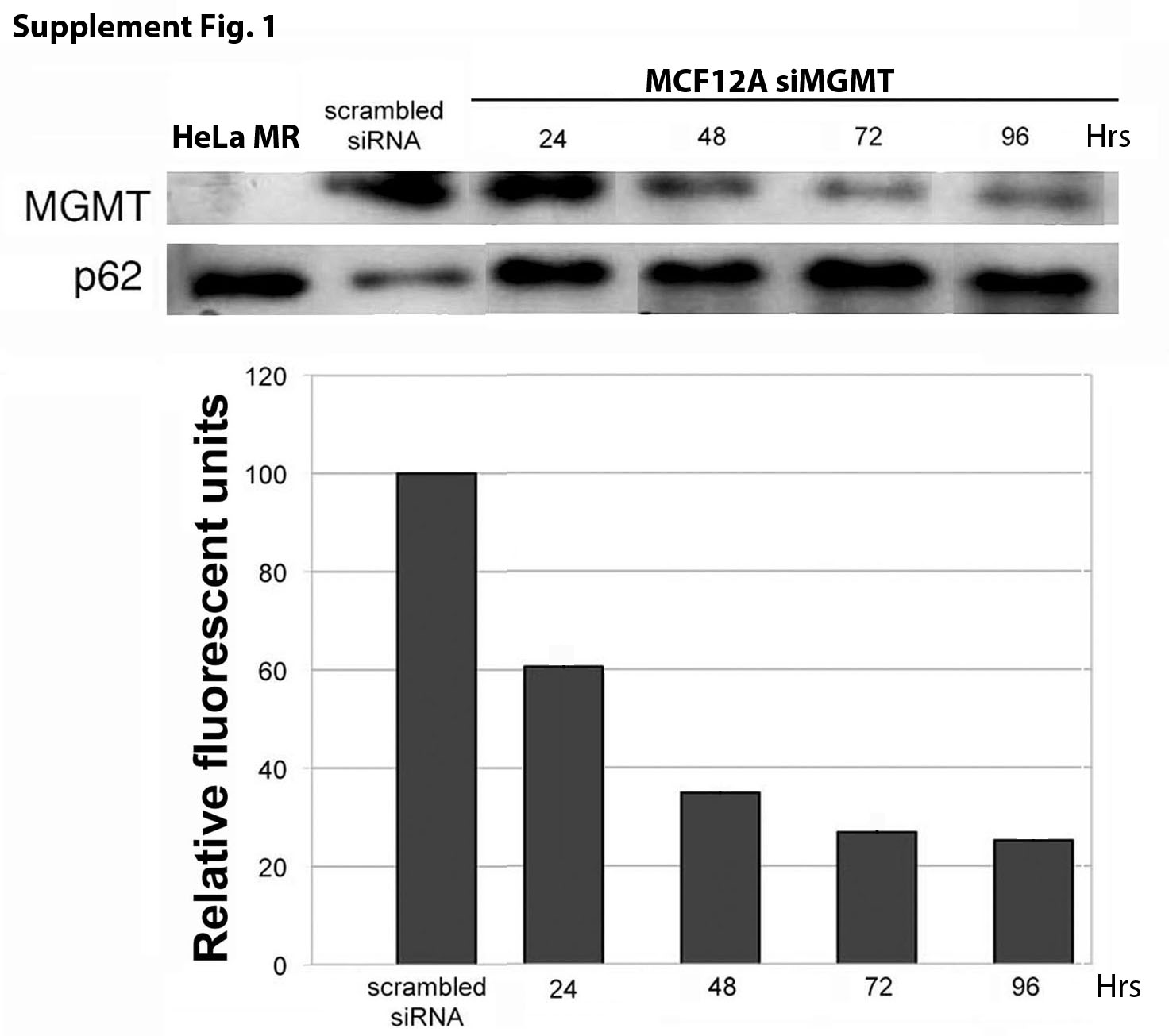

Supplement: Figure S1 — MGMT knock down in MCF12A human cells. MGMT expression was knocked down by 4 different siRNAs against MGMT. Upper figure is immunoblot of MGMT protein expression after loading equal protein concentrations in each lane, up to 96 hr after MNNG exposure with p62 as a loading control. Lower graph is a histogram produced by measurement of each fluorescent MGMT band against the p62 loading control in the same lane by Alpha lnnotech Fluorochem HD2, histograms produced by Prism GraphPad software. (ZIP) [file pone.0074071.s001.zip › Suppl 1.tif]

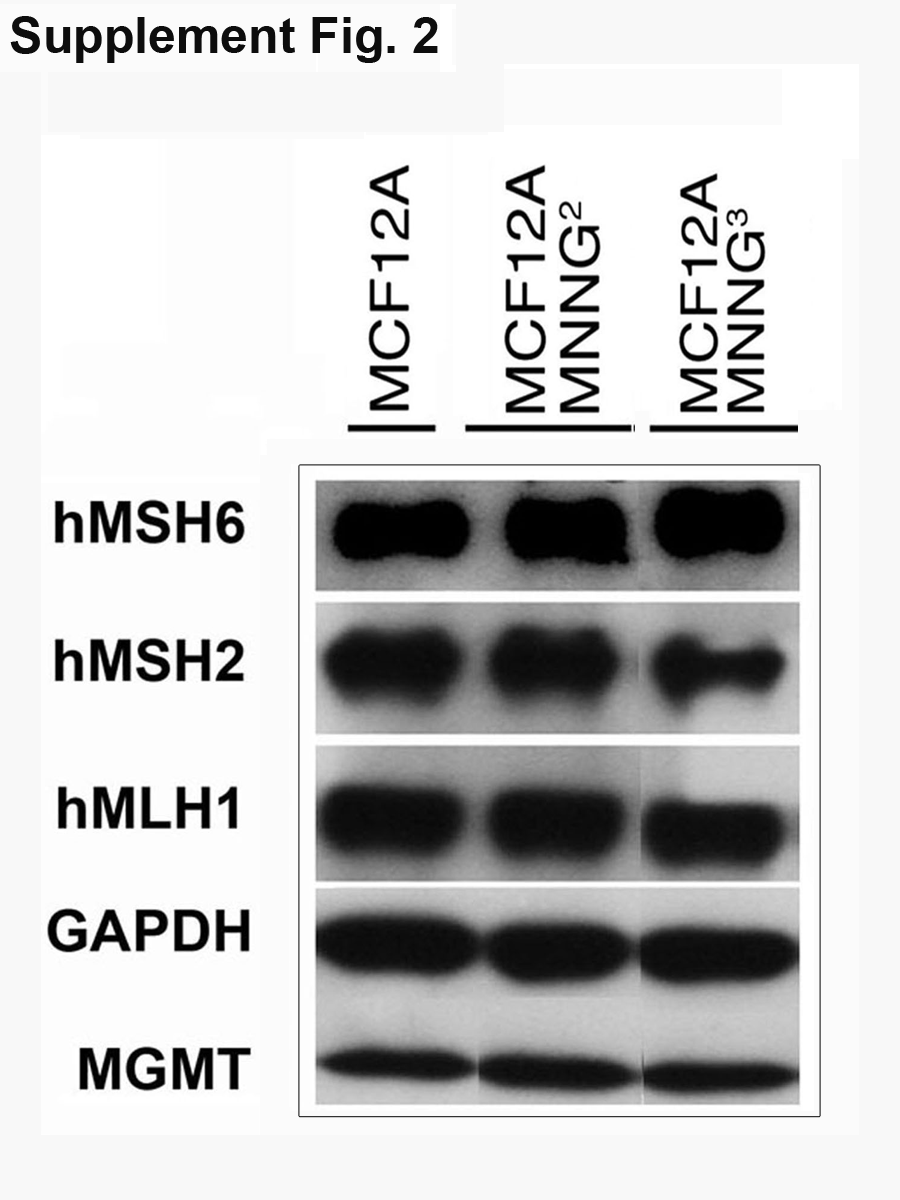

Supplement: Figure S2 — Repeated exposure to MNNG does not alter MMR or MGMT expression in surviving MCF12A populations. MMR protein expression of equal protein concentrations from MCF12A original cell line and from surviving MCF12A cells grown from three sequential MNNG exposures that result in 0% classic colony survival (8 µM). (ZIP) [file pone.0074071.s002.zip › Rev Suppl 2.tif]

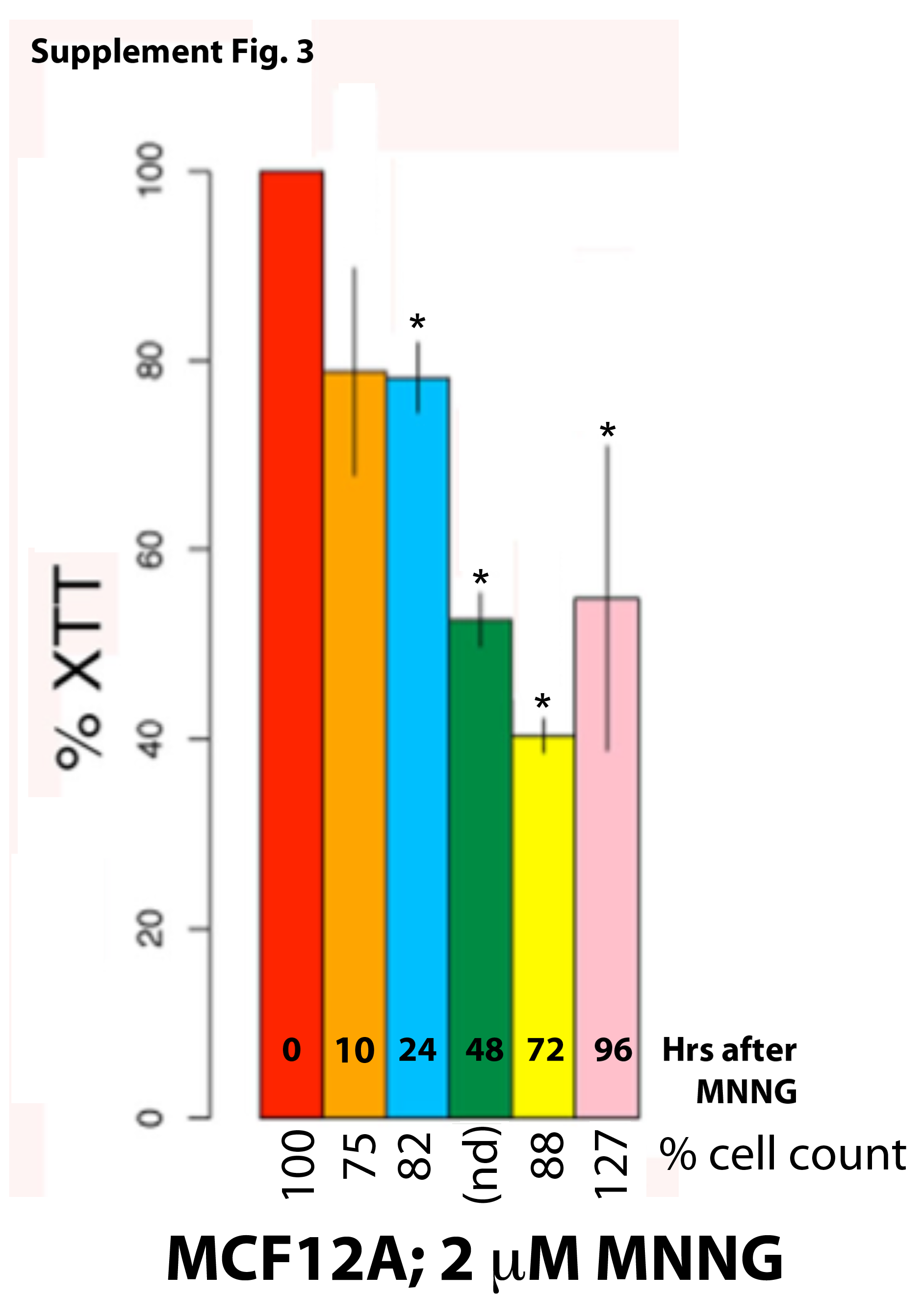

Supplement: Figure S3 — MCF12A cells exposed to 2 µM MNNG regain metabolism by 96 hr after exposure. MCF12A cells exposed to 2 µM MNNG results in 10% classic colony survival (Figure 1), allowing surviving cells to exhibit increased metabolic activity by 96 hr, although still significantly lower than 0 hr (untreated) control (compare to Figure 3A). Asterisks (*) denote statistically significant differences at P < 0.05 between the metabolic rate measured at that time point and the 0 hr (untreated) metabolic rate of each cell line. Histograms produced by Prism GraphPad software, error bars indicate SD. Statistical significance determined by student t-test using Prism GraphPad software. This experiment was performed two times. (ZIP) [file pone.0074071.s003.zip › Rev Suppl 3.tif]

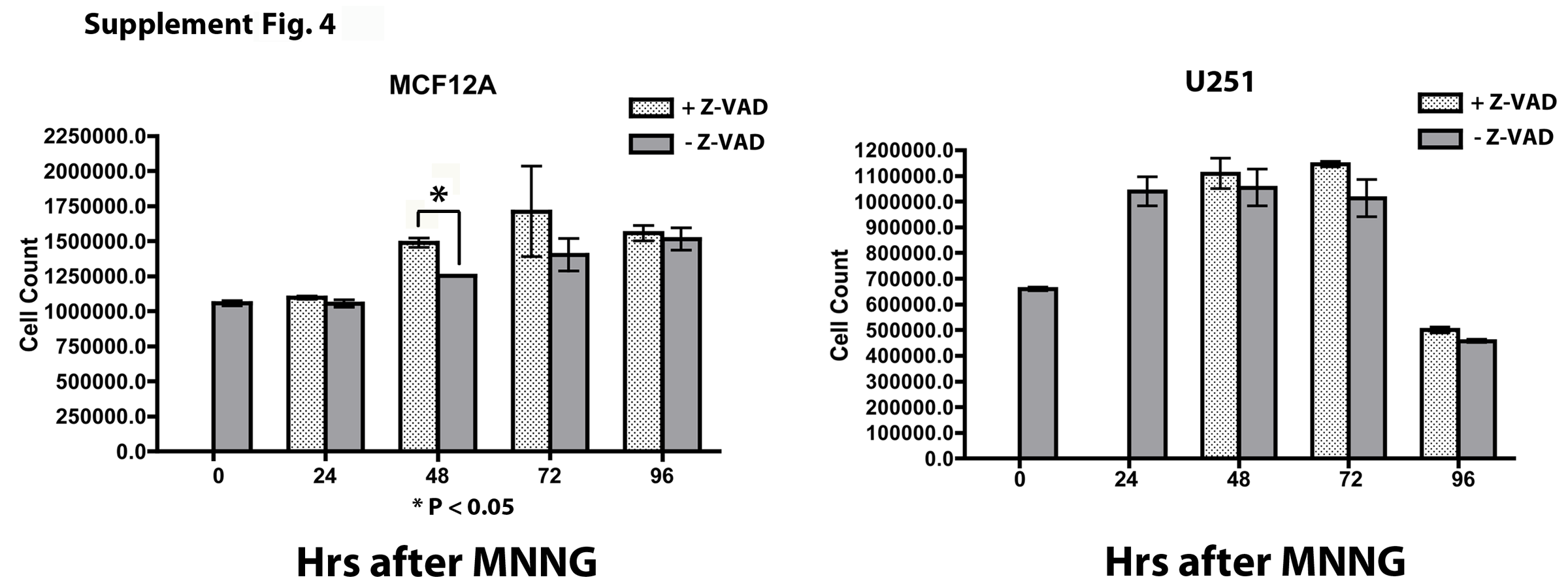

Supplement: Figure S4 — Treatment of MCF12A and U251 cells with Z-VAD decreases cell death in MCF12A cells, but not U251 cells. MCF12A cells exhibit decreased cell death at 48 hr after MNNG treatment (8 µM) by addition of Z-VAD to media. U251 cells do not exhibit decreased cell death at any time point up to 96 hr after MNNG treatment (0.2 µM). Asterisk (*) denotes statistically significant differences at P < 0.05 between the cell count measured at that time point (48 hr) and the 0 hr (untreated) MCF12A cell count. Histograms produced by Prism GraphPad software, error bars indicate SD. Statistical significance determined by student t-test using Prism GraphPad software. These experiments were performed a minimum of three times. (ZIP) [file pone.0074071.s004.zip › Rev Suppl 4.tif]
